# The socket-shield technique in orthodontics: a method for alveolar ridge preservation

**DOI:** 10.1007/s10006-025-01501-9

**Published:** 2026-01-10

**Authors:** Yuhang Zhang, Zhe Ji, Li Lin, Hui Ye, Jianxin Ji

**Affiliations:** 1 Department of Stomatology, The First Affiliated Hospital of Guangzhou Medical University, Guangzhou, Guangdong 510120 China; 2 Center for Information Technology and Statistics, The First Affiliated Hospital of Sun Yat-sen University, Guangzhou, Guangdong 510080 China; 3 Department of Stomatology, Meizhou People’s Hospital, Meizhou, Guangdong 514031 China

**Keywords:** Bone preservation, First premolar extraction, Orthodontics, Socket-shield technique

## Abstract

**Background:**

This study was conducted to investigate the clinical efficacy of Socket-Shield Technique on the adult first premolar bone preservation in Orthodontic Extraction Treatment.

**Methods:**

26 patients who were underwent Orthodontic Extraction Treatment in the First Affiliated Hospital of Guangzhou Medical University were enrolled. Extracted teeth were paired and randomly allocated into two groups: test group (Socket-Shield Technique) and control group. Finally, the socket-shield was removed when the adjacent tooth moved proximate to the shield during the process of closing orthodontic gap. Cone beam computerized tomography were utilized to access the buccolingual resorption and vertical resorption at 1 mm, 3 mm and 5 mm from a reference plane of alveolar crest, as well as tooth movement rate.

**Results:**

The first premolar alveolar bone height resorption of test group were significantly lower than those in control group. Resorption of alveolar bone width at 1 mm and 3 mm above the reference plane at T1, T2 and T3 in the test group were significantly lower than those in the control group. There were no significant differences at 5 mm above reference plane. The tooth movement rate between the test group and the control group have no significant difference.

**Conclusions:**

Socket-Shield Technique has a positive clinical effect on preserving the alveolar bone of the first premolar in adults with thin buccal alveolar bone and does not exert any discernible influence on the rate of orthodontic tooth movement in Orthodontic Extraction Treatment.

**Clinical trial registration:**

The trial was registered in Clinical Trial Registry (https://clinicaltrials.gov/) on 02/01/2024 and the registration number is NCT06510621.

**Supplementary information:**

The online version contains supplementary material available at 10.1007/s10006-025-01501-9.

## Background

In clinical orthodontic work, the necessity for extraction treatment frequently arises due to crowding or protrusion of the dentition [1]. Following the extraction procedure, the subsequent phases of orthodontic treatment, including alignment, leveling, and closing of the orthodontic gap, typically last six months to a year or even longer [2–4]. Obvious physiological alveolar bone resorption occurs after the tooth extraction, leading to a reduction in both the vertical and buccolingual (horizontal) dimensions of the alveolar bone [5, 6]. Research has shown that the mean buccal alveolar bone thickness of the maxillary and mandibular first premolars is notably thin, particularly in comparison to the thickness of second premolars [7, 8]. The resorption in the first premolars extraction area frequently manifests as a pronounced concavity on the buccal aspect [6]. Moreover, the presence of a labial frenum may impede wound healing in this region [9, 10], thereby posing challenges in the process of orthodontically relocating teeth into the extraction site. Attempts to forcefully move the teeth into that area may give rise to a spectrum of complications, including but not limited to bone dehiscences [11, 12], bone fenestration, periodontal and pulp symptoms, dental root resorption, and gingival recession [13–15].

Systematic review studies have reported an average buccolingual resorption of 1.08–1.9 mm and a vertical resorption in the range of 0.95–1.2 mm three months after extraction [16, 17]. At six months after tooth extraction, the buccolingual resorption averages within 2.63–5.1 mm, while vertical resorption averages within 0.7–1.5 mm [16, 17]. One year after extraction, the buccolingual resorption ranged from 0.4 to 6.1 mm and vertical resorption ranged from 0.4 to 4.1 mm [16]– [17]. Notably, the alveolar bone resorption mainly manifests on the buccal aspect due to the presence of thicker mucosa, rich blood vessels, and alveolar bone structures on the lingual aspect [18].

In the year 2010, Hurzeler et al. [19] introduced an approach for immediate implant surgery involving the preservation of a healthy root shield on the labial aspect. This technique aimed to retain the periodontal membrane, thereby mitigating the bone resorption. The results showed that the labial bone plate remained unaltered in terms of bone tissue reconstruction or resorption after the labial side socket-shield was preserved. Building upon this foundation, Haseeb et al. [20] conducted a study of socket-shield technique (SST) along with a modified SST which involved retaining the buccal tooth slice without implantation. Their findings concluded that both SST and modified SST were efficacious in preventing buccal bone plate resorption after tooth extraction, thereby enhancing the long-term aesthetic effect of crown and bridge restorations.

The application of SST in the field of clinical orthodontics has not been frequently reported [21, 22]. Based on the extant research and theory, this study aimed to explore the implementation of the SST within the context of orthodontic treatment involving first premolar extraction. Specifically, this study sought to investigate the clinical effects of SST on preserving the alveolar bone in the adult first premolar region of both the maxillary and mandibular jaws, aiming to provide a foundational understanding of SST’s potential application within clinical orthodontic work.

## Materials and methods

### Participants

Between January 2024 and October 2024, 28 patients, who were underwent Orthodontic Extraction Treatment in the First Affiliated Hospital of Guangzhou Medical University were enrolled. Extracted teeth were paired and randomly allocated into two groups: test group (Socket-Shield Technique) and control group.

Patients were included in the study if they reached all of the following criteria: (1) planned orthodontic treatment necessitated the extraction of the maxillary and/or mandibular first premolars bilaterally; (2) Diagnosis of maxillary or bimaxillary protrusion with crowding less then 4 mm; (3) complete permanent dentition; (4) optimal oral hygiene status without evidence of periodontal disease; (5) minimum age requirement of 18 years; (6) cone beam computerized tomography (CBCT) showed that the buccal alveolar bone thickness was less than 0.5 mm and the vertical defect was greater than 2 mm; and (7) agreed to the orthodontic extraction treatment plan and signed the treatment informed consent form and the experimental informed consent form.

The study exclusion criteria were as follows: (1) systemic comorbidities such as hypertension, heart disease, diabetes, osteoporosis, or hyperthyroidism; (2) active infection in proposed extraction sites; (3) dental root caries in the extraction area; (4) presence of buccal gingival recession in extraction sites; (5) declination of SST treatments; and (6) pregnancy or lactation.

This study was conducted in accordance with the ethical principles outlined in the Declaration of Helsinki, the Consolidates Standards of Reporting Trials (adhered to CONSORT guidelines), and the Good Clinical Practice (GCP) guideline. The protocol received formal review and approval from the Ethics Committee of the First Affiliated Hospital of Guangzhou Medical University (Approval No. 2018-27). all enrolled participants provided written informed consent for both study participation and subsequent data publication.

### Surgical procedure

**Fig. 1:**
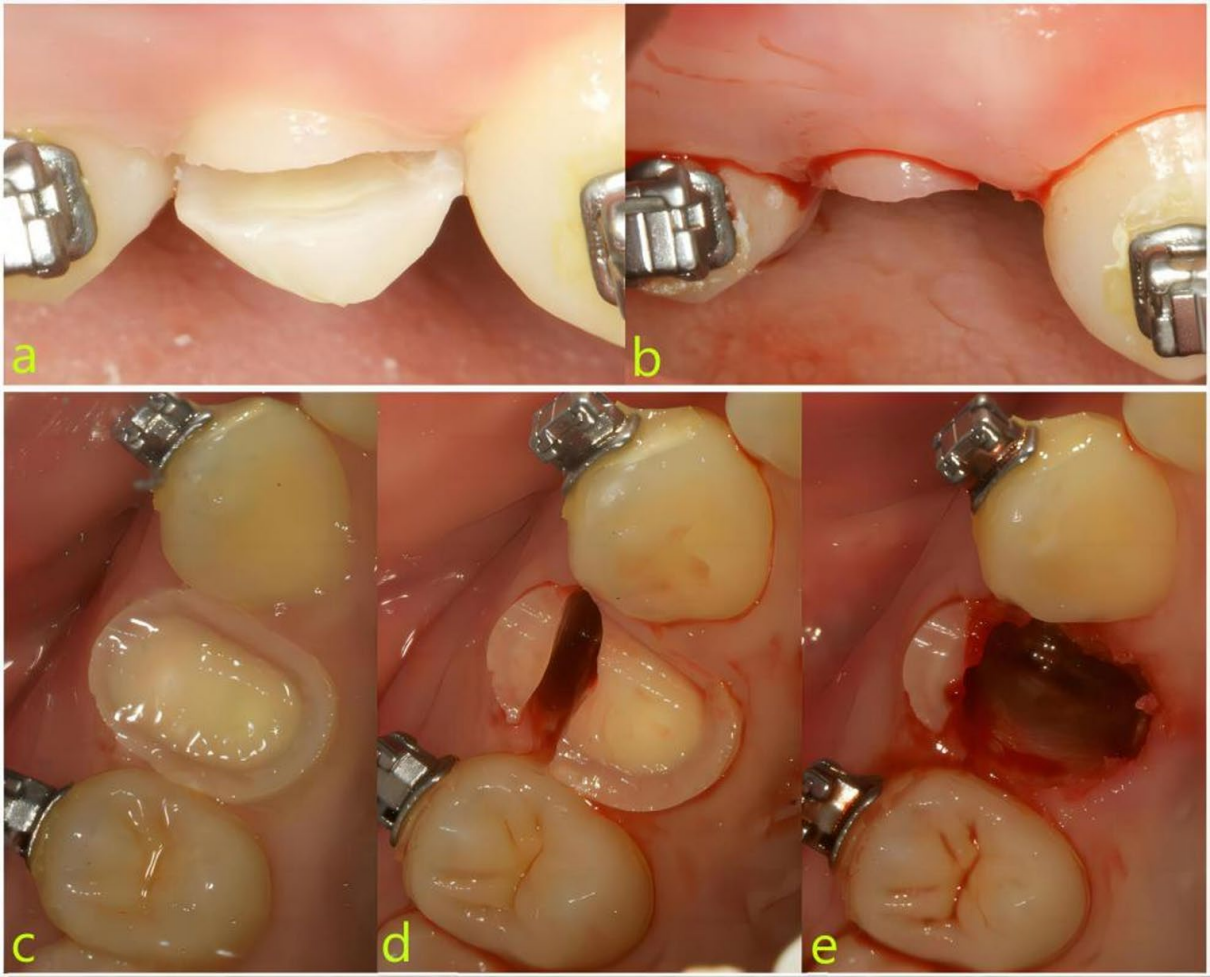
Surgical process of the test group (SST). **a** and **b**, Cut the tooth crown; **c** and **d**, divided along the long axis; **e**, trim the socket shield

**Fig. 2:**
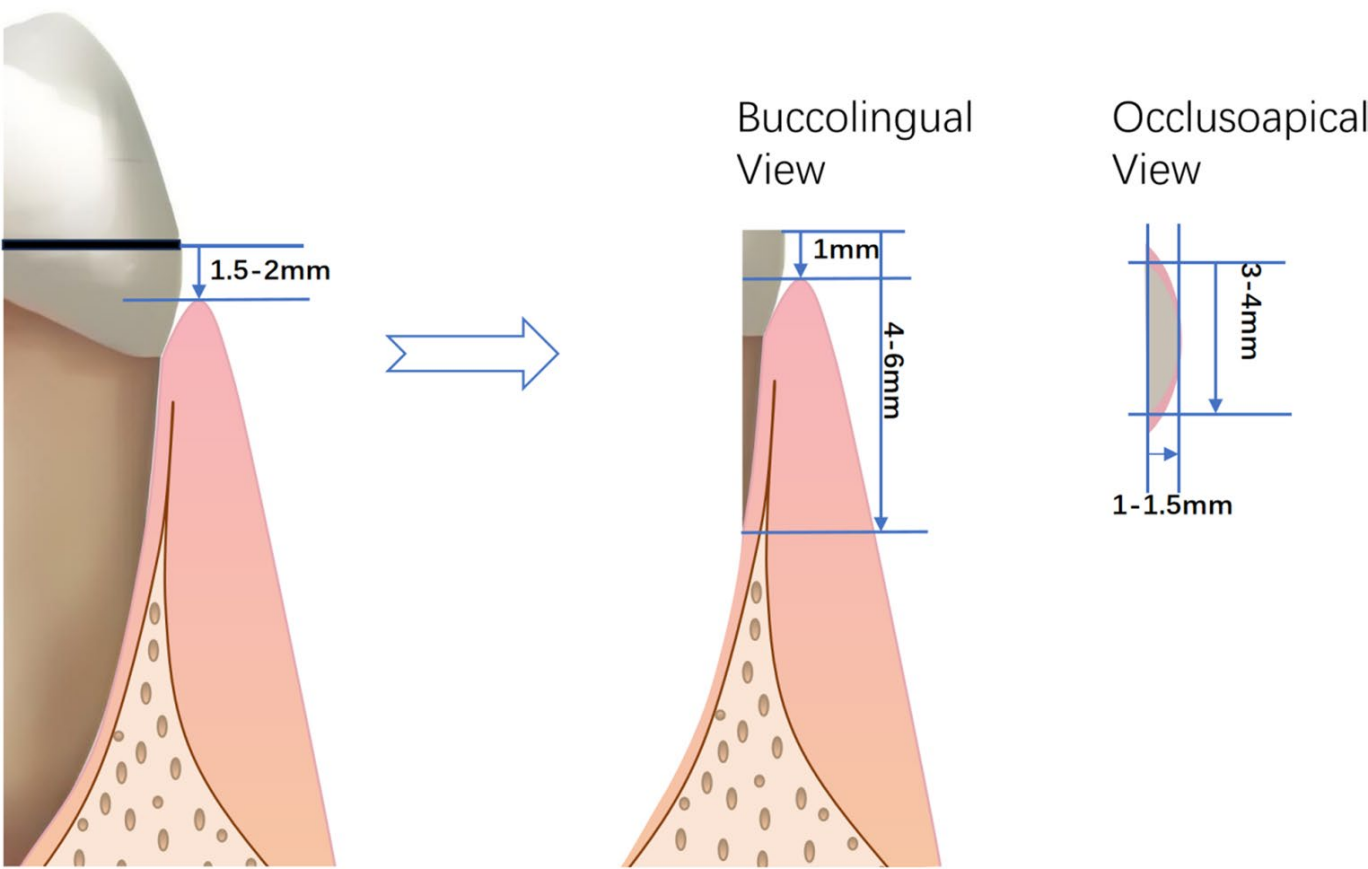
Dimensional Drawing of SST surgical process

Test Group (SST): A high-speed turbine was utilized to section the tooth crown 1.5–2 mm mm coronal to the gingival margin (Figs. 1a and b and 2). The tooth was subsequently bisected along its long axis, maintaining an approximate 2 mm distance from the buccal aspect (Figs. 1c and d and 2). The palatal roots were extracted using minimally invasive elevation and extraction forceps. The buccal socket shield was then meticulously trimmed to achieve the following dimensions: 4–6 mm in height (extending 1 mm above the buccal gingival margin) [22], 3–4 mm in width, and 1–1.5 mm in thickness [23, 24]. Trimming was performed under direct visual guidance, with periodic calibration using a periodontal probe to ensure precision (Figs. 1e and 2). The socket shield was retained until the adjacent tooth approximated the extraction site during orthodontic space closure, at which point it was removed using minimally invasive forceps.

**Fig. 3:**
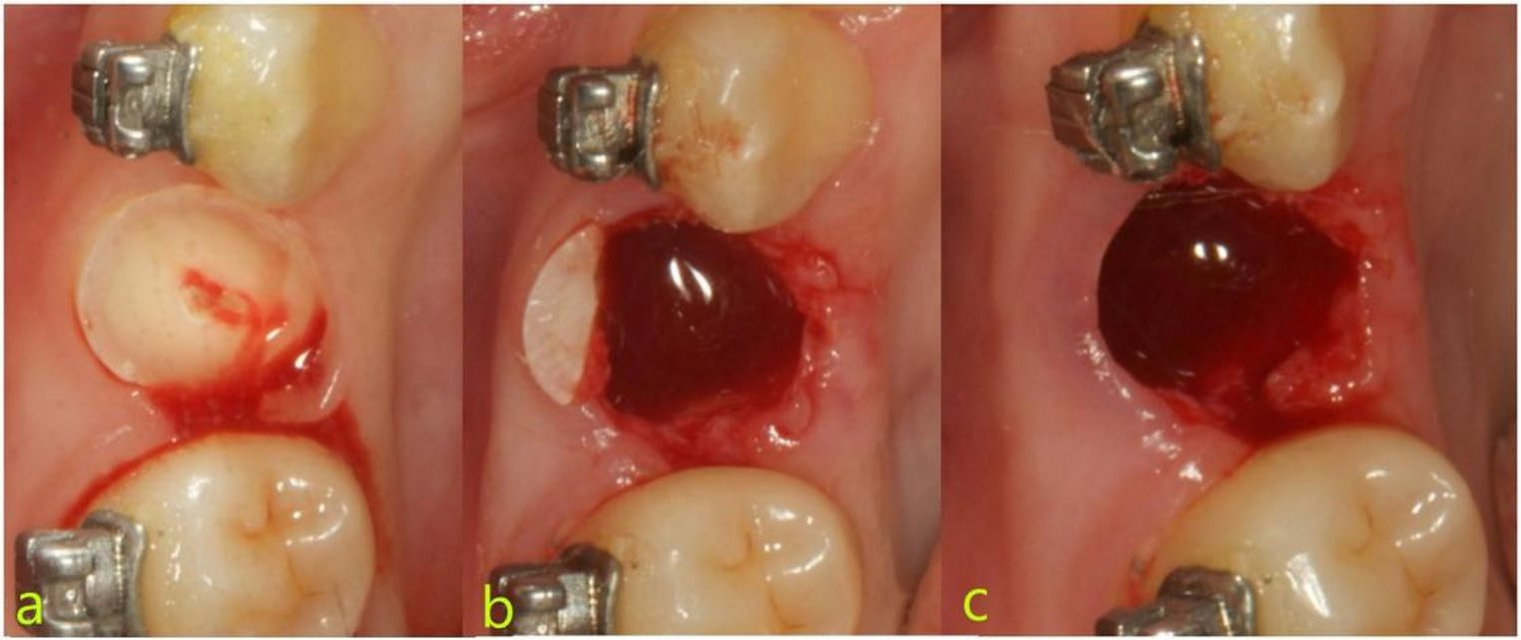
Surgical process of the control group (non-SST). **a**, Cut the tooth crown; **b**, divided the tooth and trim the socket shield; **c**, extract the shield immediately

Control Group (non-SST): The same procedural steps (crown sectioning, root division, palatal root removal, and socket shield trimming) were followed. However, in this group, the socket shield was extracted immediately after trimming (Fig. 3).

Orthodontic treatment commenced right after bonding the brackets, with tooth extraction performed immediately afterward to enable prompt application of orthodontic forces. After the tooth extraction, patients were prescribed clindamycin and ibuprofen sustained-release capsules, and instructed to use oral rinse. Additionally, oral health education was provided to ensure compliance with postoperative care. Fixed orthodontic treatment was initiated using straight wire (AO self-ligating brackets, 0.022 × 0.028 inch slot, Empower^®^ Philosophies, American Orthodontics, Sheboygan, USA). The alignment and leveling procedures phase was completed within 3 to 4 months. Subsequently, space closure was performed using a 0.019 × 0.025 inch (0.48 mm x 0.64 mm) stainless steel square wire with sliding force calibrated to deliver approximately 150 g via a force gauge.

During follow-up visits, the following parameters were assessed to evaluate the condition of socket shield: the mobility of socket-shield, and the clinical manifestations such as color, texture, and bleeding of gingiva.

### Measurement methods

To assess the clinical impact of SST versus the conventional extraction approach (non-SST) on alveolar bone width and height in the first premolar region, the left and right first premolars in same jaw were paired and randomly allocated to either the test group (SST) or the control group (non-SST). Four evaluation time points were established: prior to tooth extraction (T0), three months after tooth extraction (T1), at the time of socket-shield removal (T2), and three months post socket-shield removal (T3). CBCT scans (120 kVp and 18.66 mAs, voxel size 0.3, Alpha Plus RCT700-SC, RayScan, Hwaseong-si, Korea) were obtained at each time point.

**Fig. 4:**
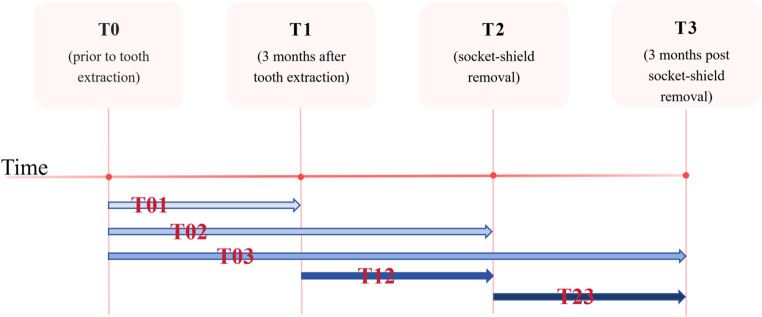
Time points and time intervals were defined for data analysis

For data analysis, the following intervals were used (Fig. 4): (1) from before tooth extraction to three months after extraction (T_01_), (2) from before tooth extraction to the time of socket-shield removal (T_02_), (3) from three months after tooth extraction to the time of socket-shield removal (T_12_), (4) from before tooth extraction to three months after socket-shield removal (T_03_), (5) from the time of socket-shield removal to three months after socket-shield removal (T_23_).

CBCT data were imported into Invivo Dental 5.0 software (Anatomage, Inc., Milan, Italy) for three-dimensional (3D) coordinate system construction and quantitative assessment of alveolar bone parameters [25].

For the mandibular measurements, the most anterior-inferior point of the left mental foramen was designated as the coordinate origin. The X-axis was defined as the parallel line connecting the most anterior-inferior points of two mental foramina, while the Y-axis was set parallel to the inferior border of the left mandible. A corresponding symmetric coordinate system was constructed for the right mandible.

For the maxilla, the origin was defined as the most anterior point of the anterior nasal spine. The X-axis was aligned parallel to the line connecting the lowest points of the bilateral maxillary sinuses, and the Y-axis was parallel to the palatal plane.

To ensure measurement reproducibility across time points, a reference plane was established at T0. This plane was defined as a cross-sectional slice parallel to the XZ plane intersecting the lowest point of the alveolar crest. At each time point (T0, T1, T2, T3), the Z-coordinate of the lowest point of the alveolar crest was recorded as alveolar bone height. Alveolar width was measured as the (X, Y)-coordinate at distances of 1 mm, 3 mm, and 5 mm apical to the reference plane. These measurements were denoted as H0/W0_(1,3,5)_, H1/W1_(1,3,5)_, H2/W2_(1,3,5)_, and H3/W3_(1,3,5)_, respectively. Changes in alveolar bone height and width were calculated as follows: ΔH1 = H1-H0/ΔW1 = W1-W0 for T_01_, ΔH2 = H2-H0/ΔW2 = W2-W0 for T_02_, and ΔH3 = H3-H0/ΔW3 = W3-W0 for T_03_.

Additionally, 3D CBCT reconstructions were utilized to determine the distance between the most convex points of the adjacent teeth in the mesial and distal distance of the orthodontic gap. These distances were recorded as D1, D2, and D3 at T1, T2, and T3, respectively. The rate of tooth movement was calculated by dividing the change in tooth position (mm) by the duration of the respective intervals (months) [26]: (D1-D2)/(T2-T1) for T_12_ and (D2-D3)/(T3-T2) for T_23_.

All measurement were independently performed by two clinicians who underwent standardized calibration and training. The reliability of the measurements was assessed using the intraclass correlation coefficient (ICC). Only data with an ICC ≥ 0.75 were considered acceptable, in which case the average of the two observers’ measurements was used for final analysis.

### Statistical analysis

All data were analyzed by SPSS 20.0 statistical software. The S-W test and F-test were employed to verify whether the data were normally distributed and whether the variances were homogeneous. The data from both the test group and the control group were evaluated using paired samples t-test. A significance level of *P* < 0.05 was considered statistically significant.

## Results

**Fig. 5:**
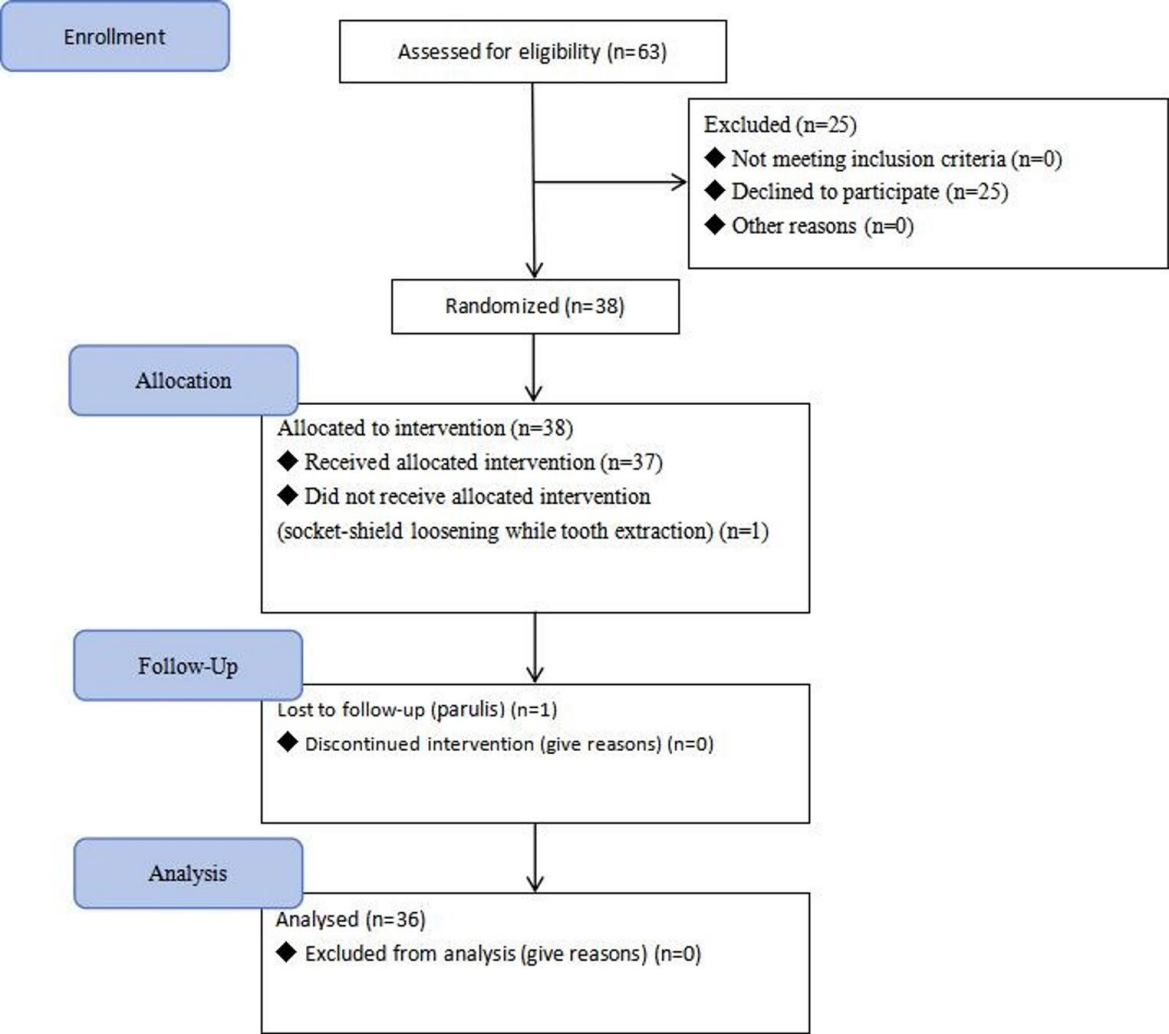
Case inclusion flowchart. (n) representing the total pairs of single-jaw

Among the initial 28 patients (38 single-jaw) that were selected in this study. one patient was lost to follow-up due to socket-shield loosening during tooth extraction, and one patient was excluded due to both socket-shield loosening and the development of parulis during postoperative follow-up. Ultimately, 26 patients (12 males, 46%; 14 females, 54%) were included (Fig. 5)., with an overall mean age of 28.4 ± 9.3 (range 18–51), including 10 double-jaw and 16 single-jaw (a total of 36 single-jaw and 72 tooth extraction sites).

Two female patients (2/26 patients, 2/72 sites) experienced obvious pain and chills in the extraction area after tooth extraction. Additionally, One patient (1/26 patients, 1/72 sites) had more bleeding than usual, potentially due a minor blood vessel rupture during tooth extraction. To address the bleeding, a Gelatin spring was inserted into the extraction socket, and pressure was applied with a cotton ball to the surgical area for thirty minutes, resulting in the cessation of bleeding. These complications did not significantly impact the patients’ daily activities. The average retention time of the socket-shield was 5.89 ± 1.24 months (95% CI 5.49–6.29).

Alveolar bone height analysis showed significantly less bone loss in the test group than in the control group at all time points (T01, T12, and T23; *P* < 0.05; Table 1), demonstrating consistent vertical bone preservation throughout the study.

**Table 1:** Vertical alveolar bone loss ($\overline x\pm S$)

Vertical alveolar bone loss	Control Group (mm)	Test Group (mm)	t	*P*
T_01_ (△H1)	1.07 ± 0.32 (95% CI 0.97–1.17)	0.28 ± 0.07 (95% CI 0.26–0.31)	15.08	< 0.001*
T_02_ (△H2)	1.33 ± 0.34 (95% CI 1.22–1.44)	0.35 ± 0.08 (95% CI 0.32–0.37)	17.65	< 0.001*
T_03_ (△H3)	1.43 ± 0.37 (95% CI 1.31–1.56)	0.38 ± 0.08 (95% CI 0.35–0.40)	17.91	< 0.001*

The time points prior to tooth extraction, three months after tooth extraction, when the socket-shield extracted, and three months after socket-shield extraction were marked as T0, T1, T2, and T3 respectively. T01 (△H1) was the height of the alveolar bone resorption from T0 to T1, T02 (△H2) was the amount of alveolar bone height resorption from T0 to T2, and T03 (△H3) was the amount of alveolar bone resorption from T0 to T3. *: *P* < 0.05, CI: Confidence interval

For buccolingual bone dimensions, the test group exhibited significantly reduced bone width loss at 1 mm and 3 mm from the reference plane (*P* < 0.05; Tables 2 and 3), indicating a pronounced coronal preservation effect.

**Table 2:** Buccolingual alveolar bone loss at 1 mm from the reference plane ($\overline x\pm S$)

Buccolingual alveolar bone loss	Control Group (mm)	Test Group (mm)	t	*P*
T_01_ (△W1)	2.14 ± 0.58 (95% CI 1.95–2.33)	1.17 ± 0.31 (95% CI 1.07–1.27)	8.72	< 0.001*
T_02_ (△W2)	3.22 ± 0.80 (95% CI 2.96–3.48)	1.38 ± 0.30 (95% CI 1.28–1.48)	12.64	< 0.001*
T_03_ (△W3)	3.47 ± 0.79 (95% CI 3.21–3.73)	1.47 ± 0.31 (95% CI 1.37–1.57)	13.75	< 0.001*

The time points prior to tooth extraction, three months after tooth extraction, when the socket-shield extracted, and three months after socket-shield extraction were marked as T0, T1, T2, and T3 respectively. T01 (△W1) was the amount of alveolar bone width resorption from T0 to T1, T02 (△W2) was the amount of alveolar bone width resorption from T0 to T2, and T03 (△W3) was the amount of alveolar bone width resorption from T0 to T1. *: *P* < 0.05, CI: Confidence interval

**Table 3:** Buccolingual alveolar bone loss at 3 mm from the reference plane ($\overline x\pm S$)

Buccolingual alveolar bone loss	Control Group (mm)	Test Group (mm)	t	*P*
T_01_ (△W1)	1.67 ± 0.61 (95% CI 1.47–1.87)	0.93 ± 0.32 (95% CI 0.83–1.03)	7.29	< 0.001*
T_02_ (△W2)	1.96 ± 0.66 (95% CI 1.75–2.18)	1.06 ± 0.32 (95% CI 0.96–1.17)	7.70	< 0.001*
T_03_ (△W3)	2.09 ± 0.66 (95% CI 1.87–2.30)	1.12 ± 0.32 (95% CI 1.01–1.21)	8.11	< 0.001*

The time points prior to tooth extraction, three months after tooth extraction, when the socket-shield extracted, and three months after socket-shield extraction were marked as T0, T1, T2, and T3 respectively. T01 (△W1) was the amount of alveolar bone width resorption from T0 to T1, T02 (△W2) was the amount of alveolar bone width resorption from T0 to T2, and T03 (△W3) was the amount of alveolar bone width resorption from T0 to T1. *: *P* < 0.05, CI: Confidence interval

However, at 5 mm from the reference plane, no significant difference was observed between groups (*P* > 0.05; Table 4), suggesting that the intervention’s benefit was confined mainly to the coronal region of the alveolar ridge.

**Table 4:** Buccolingual alveolar bone loss at 5 mm from the reference plane ($\overline x\pm S$)

Buccolingual alveolar bone loss	Control Group (mm)	Test Group (mm)	t	*P*
T_01_ (△W1)	0.54 ± 0.29 (95% CI 0.44–0.63)	0.48 ± 0.22 (95% CI 0.41–0.55)	1.28	0.21
T_02_ (△W2)	0.65 ± 0.30 (95% CI 0.55–0.75)	0.58 ± 0.23 (95% CI 0.50–0.65)	1.59	0.12
T_03_ (△W3)	0.69 ± 0.32 (95% CI 0.59–0.79)	0.59 ± 0.23 (95% CI 0.52–0.67)	1.98	0.07

The time points prior to tooth extraction, three months after tooth extraction, when the socket-shield extracted, and three months after socket-shield extraction were marked as T0, T1, T2, and T3 respectively. T01 (△W1) was the amount of alveolar bone width resorption from T0 to T1, T02 (△W2) was the amount of alveolar bone width resorption from T0 to T2, and T03 (△W3) was the amount of alveolar bone width resorption from T0 to T1.

In addition, the potential impact of the intervention on orthodontic tooth movement was examined. As shown in Table 5, no significant difference in tooth movement rate was detected between the test and control groups (*P* > 0.05; Paired Samples T-Test). These results indicate that while the intervention effectively reduced bone resorption, it did not alter the rate of orthodontic tooth movement.

**Table 5:** Orthodontic tooth movement rate in T_12_ and T_23_ ($\overline x\pm S$)

Teeth movement rate	Control Group (mm/month)	Test Group (mm/month)	t	*P*
T_12_ (D1-D2)/(T2-T1)	1.10 ± 0.25 (95% CI 1.01–1.18)	1.13 ± 0.28 (95% CI 1.04–1.22)	−0.17	0.10
T_23_ (D2-D3)/(T3-T2)	1.11 ± 0.26 (95% CI 102 − 1.19)	1.15 ± 0.24 (95% CI 1.07–1.23)	−1.90	0.07

The time points prior to tooth extraction, three months after tooth extraction, when the socket-shield extracted, and three months after socket-shield extraction were marked as T0, T1, T2, and T3 respectively. T12 (D1-D2)/(T2-T1) is the average tooth movement rat from T1 to T2 (mm/month), and T23 (D2-D3)/(T3-T2) is the average tooth movement rat from T2 to T3.

## Discussion

In this study, vertical alveolar bone height loss at the first-premolar site was significantly lower in the test group compared with the control group. Similarly, buccolingual bone width reduction at 1 mm and 3 mm above the reference plane at T1, T2, and T3 was markedly less in the test group, whereas no significant differences were observed at the 5-mm level. Additionally, the rate of orthodontic tooth movement did not differ significantly between the two groups.

According to Schropp’s research [27], buccolingual alveolar bone resorption measured 3.8 mm after a three-month period, followed by a further resorption to 5.1 mm after six months, and culminating to a peak of 6.1 mm after twelve months. Concurrently, Tan WL’s [28] study indicated that the buccolingual resorption of alveolar bone after tooth extraction accounted for 32% of the total alveolar bone width after three months, with further reductions ranging from 29% to 63% after six months. Based on the aforementioned research, this study sought to investigate and quantify the variations in both alveolar bone height and width at four distinct time points: prior to tooth extraction (T0), three months after tooth extraction (T1), at the juncture of socket-shield extraction which occurring at an average of 5.89 ± 1.24 months after tooth extraction (T2), and three months after socket-shield extraction (T3).

Alveolar bone resorption after tooth extraction primarily occurred on the buccal aspect of the alveolar ridget [18], characterized by a sloping shape with a narrow ridge and a wide base. Presently, majority studies measure the alveolar bone width at specific intervals of 1 mm, 3 mm, and 5 mm from the alveolar ridge to evaluate the effectiveness of bone preservation [29, 30]. Additionally, the buccal socket-shield, with a height typically ranging from 4 to 6 mm, had an apex approximately 3 mm away from the alveolar ridge. This area is considered as technically sensitive region for surgical operation, as improper procedures may result in perforation of the buccal bone plate. Therefore, this study defined a reference plane using the lowest point of the alveolar ridge at T0 and conducted the assessment of buccolingual alveolar bone loss at 1 mm, 3 mm, and 5 mm from the reference plane.

The buccal and labial bone plates are known for their relatively thin morphology and are commonly susceptible to bone dehiscence and bone fenestration. The findings of this study demonstrated that socket-shield had a buccolingual preserving effect on alveolar bone, specifically evident at distances of 1 mm and 3 mm from the reference plane. This observation suggests that SST effectively reduced the risk of bone dehiscence occurring at the orthodontic extraction site. Conversely, at 5 mm from the reference plane, socket-shield had no significant influence on the alveolar bone, thereby indicating that SST does not impact the risk of bone fenestration. Furthermore, the buccolingual alveolar bone loss of test group at 1 mm was 1.17 ± 0.31 at T_01_ interval (three months after tooth extraction), which was similar to what was reported in Botticelli D’s research, where the buccolingual loss of alveolar bone on the lingual aspect measured 1.1 ± 0.8 mm four months after immediate implantation [31]. This alignment in results suggests that the primary buccolingual loss of alveolar bone within the test group primarily occurred on lingual aspect, with minimal loss occurring on the buccal aspect. This outcome was consistent with the data supporting the application of SST in the context of immediate implantation [32].

The findings derived from of this study showed that the test group had less alveolar bone height loss in comparison to the control group, indicating the efficacy of socket-shield in preserving alveolar bone height. Consequently, it reduced the complications associated with the reduction of bone mass at the tooth extraction site, such as bone dehiscence, root resorption, and cortical bone resistance. Within the test group, the vertical alveolar bone loss at T_02_ interval was 0.35 mm, closely aligning with the recorded of vertical loss of 0.31 mm in immediate implantation with SST six months post-operation [33]. This consistency underscores the theoretical premise that the application of SST should theoretically maintain the height of the alveolar bone [19]. Nonetheless, the results of this clinical experiment reported a slight vertical bone resorption within the test group, which may potentially be attributed to factors such as gingiva and/or alveolar bone trauma, changes in blood supply or the phenomenon of regional accelerated phenomenon after trauma. It is noteworthy, however, that this minimal loss did not exert any discernible influence on the clinical outcomes of orthodontics treatment.

In contrast to the use of SST in implantation, this study involved the extraction of the socket-shield when the adjacent tooth had migrated proximate to the shield. There was no significant difference in alveolar bone height and width loss at T_23_ interval, indicating that the extraction of socket-shield did not engender any detrimental effects on the bone preservation at the site. Three factors may account for this phenomenon. First, the remodeling of new bone within the extraction site had mostly been completed within a time-frame of 28–35 days after tooth extraction [33]. Second, the presence of the canine tooth root provided a degree of structural support to the alveolar bone as it moved towards the extraction site. Third, by the time of socket-shield extraction, the extraction site had already undergone the maturation of cancellous bone. Notably, the thickness of socket-shield typically ranged from 1 to 1.5 mm, primarily functioning as a supportive structure, while the cancellous bone on the palate aspect provided cytokines, osteoblasts, and blood supply to promote bone formation. Collectively, these factors appear to have contributed to the preservation of buccal alveolar bone.

Recent clinical orthodontic cases have sparked debates concerning the impact of bone preservation technique on the rate of orthodontic tooth movement. Some studies investigated that, after tooth extraction, the application of bone preservation through guided bone regeneration (GBR) was able to reduce alveolar bone loss. However, it has been suggested that such preservation techniques might also slow down the rate of closing orthodontic gap, thereby prolonging the overall treatment duration [34]. Conversely, alternative studies have found that the bone graft material utilized for bone preservation primarily functions as structural support and eventually undergo gradual replacement by autologous bone. This replacement process leads to higher bone density at the preservation site, potentially influencing the bone remodeling and tooth movement rate [35]. Nonetheless, certain studies have claimed that GBR bone preservation has no effect on tooth movement rate [36], as long as immediate force is applied to move the teeth after bone preservation. In this study, there was no statistically significant difference in tooth movement rate between the test group and the control group, indicating that SST had neither positive nor negative effects on tooth movement, thus presenting an advantages in orthodontic treatment. It is worth noting that this outcome diverges from prior studies employing GBR technique, possibly attributable to the absence of bone graft material in the SST approach. However, further investigation is warranted to elucidate the nuances of this distinction.

In this study, one patient was excluded due to socket-shield loosening. Subsequent examination revealed the presence of numerous debris and dental calculus alongside observable signs of surrounding swollen gingiva, indicating a poor oral hygiene practices. It is noteworthy that some scholars suggested that the socket-shield should ideally be retained at a distance of 0.5–2 mm above the alveolar ridge [23, 24]. However, in this study, in order to stabilize the gingiva, the decision was made to maintain the socket-shield to be retained approximately 1 mm above the gums, inherently significantly influenced by oral hygiene.

The present study has several limitations. First, the lack of a conventional extraction control group confines the comparison to two SST variants, with or without root-shield retention, thereby limiting the applicability of the findings and leaving the relative efficacy of SST versus traditional extraction unresolved. Additional histological studies are needed to further substantiate the observed bone-preservation effects. Future investigations should expand the sample size, incorporate stratified subgroups (e.g., age, jaw region, sex), and extend follow-up to better evaluate orthodontic space closure. The inherent visibility of the shield within the socket also complicates allocation concealment, posing challenges for blinded randomized trials.

To minimize confounding, adolescents undergoing active craniofacial growth were excluded; thus, the conclusions apply only to adults. Moreover, SST, particularly when retaining the shield, is technique-sensitive and requires dedicated training despite the presence of standardized protocols.

## Conclusion

The findings of this study collectively indicate that SST is a reliable technique for preserving alveolar bone in adult patients with thin buccal plates in the first-premolar region. SST effectively mitigates both buccolingual and vertical bone resorption following extraction, thereby maintaining the structural integrity of the alveolar ridge. Importantly, the technique does not appear to alter the rate of orthodontic tooth movement, nor does it extend the overall treatment duration. Taken together, these results suggest that SST offers a biologically advantageous and orthodontically compatible approach for managing extraction sites in cases with thin alveolar bone, supporting its potential value as an adjunctive procedure in interdisciplinary treatment planning.

## Supplementary information

Below is the link to the electronic supplementary material.

ESM 1 (DOCX 15.9 KB)

## Data Availability

The datasets used and/or analyzed during the current study are available from the corresponding author on reasonable request.
