# Supplementary material for: The socket-shield technique in orthodontics: a method for alveolar ridge preservation
Source: Oral Maxillofac Surg. 2026 Jan 10;30(1):16. doi: 10.1007/s10006-025-01501-9 (PMC12789239; doi:10.1007/s10006-025-01501-9)
Supplement: Supplementary file 1 — (DOCX 15.9 KB) [file 10006_2025_1501_MOESM1_ESM.docx]

Supplementary Table

Demographic data

| Patient No. | Age | Sex | Test group site (SST) | Control group site (non-SST) |
| --- | --- | --- | --- | --- |
| 1 | 19 | Female | 14 | 24 |
| 2 | 21 | Female | 24 | 14 |
|  |  |  | 34 | 44 |
| 3 | 18 | Female | 24 | 14 |
| 4 | 18 | Female | 24 | 14 |
|  |  |  | 34 | 44 |
| 5 | 31 | Female | 24 | 14 |
|  |  |  | 44 | 34 |
| 6 | 49 | Female | 14 | 24 |
|  |  |  | 44 | 34 |
| 7 | 51 | Female | 24 | 14 |
|  |  |  | 34 | 44 |
| 8 | 34 | Female | 24 | 14 |
|  |  |  | 44 | 34 |
| 9 | 40 | Female | 14 | 24 |
|  |  |  | 44 | 34 |
| 10 | 25 | Male | 24 | 14 |
|  |  |  | 44 | 34 |
| 11 | 18 | Male | 14 | 24 |
| 12 | 24 | Male | 44 | 34 |
| 13 | 28 | Male | 14 | 24 |
|  |  |  | 34 | 44 |
| 14 | 21 | Male | 24 | 14 |
| 15 | 32 | Male | 14 | 24 |
|  |  |  | 44 | 34 |
| 16 | 42 | Male | 14 | 24 |
| 17 | 22 | Male | 24 | 14 |
| 18 | 25 | Male | 24 | 14 |
| 19 | 31 | Male | 14 | 24 |
| 20 | 34 | Male | 14 | 24 |
| 21 | 36 | Male | 24 | 14 |
| 22 | 23 | Female | 14 | 24 |
| 23 | 25 | Female | 24 | 14 |
| 24 | 29 | Female | 14 | 24 |
| 25 | 19 | Female | 14 | 24 |
| 26 | 22 | Female | 24 | 14 |
